# Averting older adults' memory function decline via meaningful activities: a follow-up longitudinal study

**DOI:** 10.1007/s41999-024-01044-4

**Published:** 2024-09-06

**Authors:** Shoma Akaida, Takayuki Tabira, Mana Tateishi, Daijo Shiratsuchi, Suguru Shimokihara, Ryota Kuratsu, Yoshihiko Akasaki, Yuma Hidaka, Hyuma Makizako

**Affiliations:** 1 Graduate School of Health Sciences, Kagoshima University, Kagoshima, Japan; 2 Department of Physical Therapy, School of Health Sciences, Faculty of Medicine, Kagoshima University, Kagoshima, Japan; 3 Department of Occupational Therapy, School of Health Sciences, Faculty of Medicine, Kagoshima University, Kagoshima, Japan; 4 Department of Epidemiology of Aging, National Center for Geriatrics and Gerontology, Aichi, Japan; 5 Department of Occupational Therapy, School of Health Sciences, Sapporo Medical University, Hokkaido, Japan; 6 Research Fellowship for Young Scientists, Japan Society for the Promotion of Science, Tokyo, Japan

**Keywords:** Meaningful activity, Cognition, Performance, Aging, Healthy aging

## Abstract

**Purpose:**

The purpose of the study was to investigate the effects of satisfaction with meaningful activities and their performance on the memory function among community-dwelling older adults 3 years later.

**Methods:**

A longitudinal analysis was conducted on 288 community-dwelling older adults aged 65 years and more who had completed the survey in 2019 and 2022 as participants in the Tarumizu Study. Meaningful activities were assessed in 2019 using the Aid for Decision-Making in Occupation Choice for activities. Satisfaction with and performance of meaningful activities were assessed, and the bottom 25% were classified into two groups: low satisfaction and low performance. Memory function was assessed using the National Center for Geriatrics and Gerontology-Functional Assessment Tool in 2019 and 2022. Changes in memory function over 3 years were analyzed by satisfaction and performance of meaningful activities.

**Results:**

Two-way analysis of variance with group (satisfaction with and performance of meaningful activities) and time (2019 and 2022) as factors showed no statistically significant group-time interaction for memory in the satisfaction with meaningful activities group (*F* = 1.4, *p* = 0.24). Meanwhile, a statistically significant group-time interaction was observed for memory in the meaningful activity performance group, indicating that high performance of meaningful activities may play a protective role against memory decline (*F* = 7.4, *p* = 0.007).

**Conclusion:**

Higher performance of meaningful activities may protect against age-related changes in memory. A careful assessment of meaningful activities, and engagement that enhances performance may be useful in designing health support against memory decline.

**Supplementary Information:**

The online version contains supplementary material available at 10.1007/s41999-024-01044-4.

## Key summary points

**Aim:**

To investigate whether the effects of satisfaction with meaningful activities and their performance on the memory function among community-dwelling older adults three years later.

**Findings:**

This longitudinal study suggests that high performance, regardless of category of meaningful activity, is protective against changes in memory function 3 years later.

**Message:**

Engagement that uses objective assessment tools to evaluate the performance of meaningful activities and to improve performance would be useful in designing health support for memory decline.

**Supplementary Information:**

The online version contains supplementary material available at 10.1007/s41999-024-01044-4.

## Introduction

Approximately 55 million people worldwide live with dementia, with approximately 10 million cases each year [1]. This number is expected to increase to more than 150 million by 2050, highlighting the need for public health measures [2]. Dementia is preceded by a decline in cognitive function beyond that expected during normal aging [3]. Therefore, preventing cognitive decline, which is the preliminary stage of dementia, is an important aspect while considering support measures for dementia prevention [3]. To achieve the World Health Organization’s (WHO) Healthy Aging, protective factors against dementia and cognitive decline can be considered [4]. Research suggests that encouraging personal lifestyle changes, such as some physical activity and dietary control, may help protect against cognitive decline in older adults [3]. When considering such protective factors, it is important to consider meaningful activities tailored to the individual’s subjective viewpoint [5]. However, most studies have examined these activities as general activities, common to everyone (e.g., exercise activities and dietary management), and have not examined whether they are meaningful activities that reflect an individual’s subjective perspective [3].

Meaningful activities are defined as “physical, social, and leisure activities tailored to the needs and preferences of the individual” [6]. Further conceptual analysis of meaningful activities reveals five attributes: a) enjoyable, b) appropriate for personal skills, abilities, and preferences, c) relevant to personally relevant goals, d) engaging, and e) related to aspects of identity [7]. Thus, meaningful activities are interpreted as activities that reflect the subjective viewpoint of “for the individual” [8]. These activities cover diverse activities from basic ones related to daily living (eating, moving, dressing, etc.) to higher level ones, such as gardening and working [9, 10]. Meaningful activities are evaluated using subjective indicators (e.g., satisfaction and performance), which may provide a better understanding of an individual’s life [11, 12]. In older adults, satisfaction with and the performance of meaningful activities are reportedly associated with depressive symptoms and frailty [13, 14]. This suggests the importance of considering the satisfaction with and performance of meaningful activities when providing health support tailored to older adults' individual needs.

Previous studies are scattered, with reports suggesting a link between meaningful activity and cognitive function. For example, while engaging in an activity judged as meaningful (listening to pleasant music of one’s own choice), the activation of the anterior cingulate cortex was observed using Positron Emission Tomography [15, 16]. The cingulate cortex is closely related to the limbic system, and is involved in emotion, behavior, and memory [17].

Thus, meaningful activities and cognitive functions may be related [15]. However, the effects of meaningful activities on age-related changes in cognitive function remain unclear. Particularly, preventive insights into memory function, a strong predictor of the development of dementia, are of particular importance [18]. Therefore, this study investigated the effects of satisfaction with and performance of meaningful activities on the memory function of community-dwelling older adults after 3 years.

## Method

### Participants

**Fig. 1:**
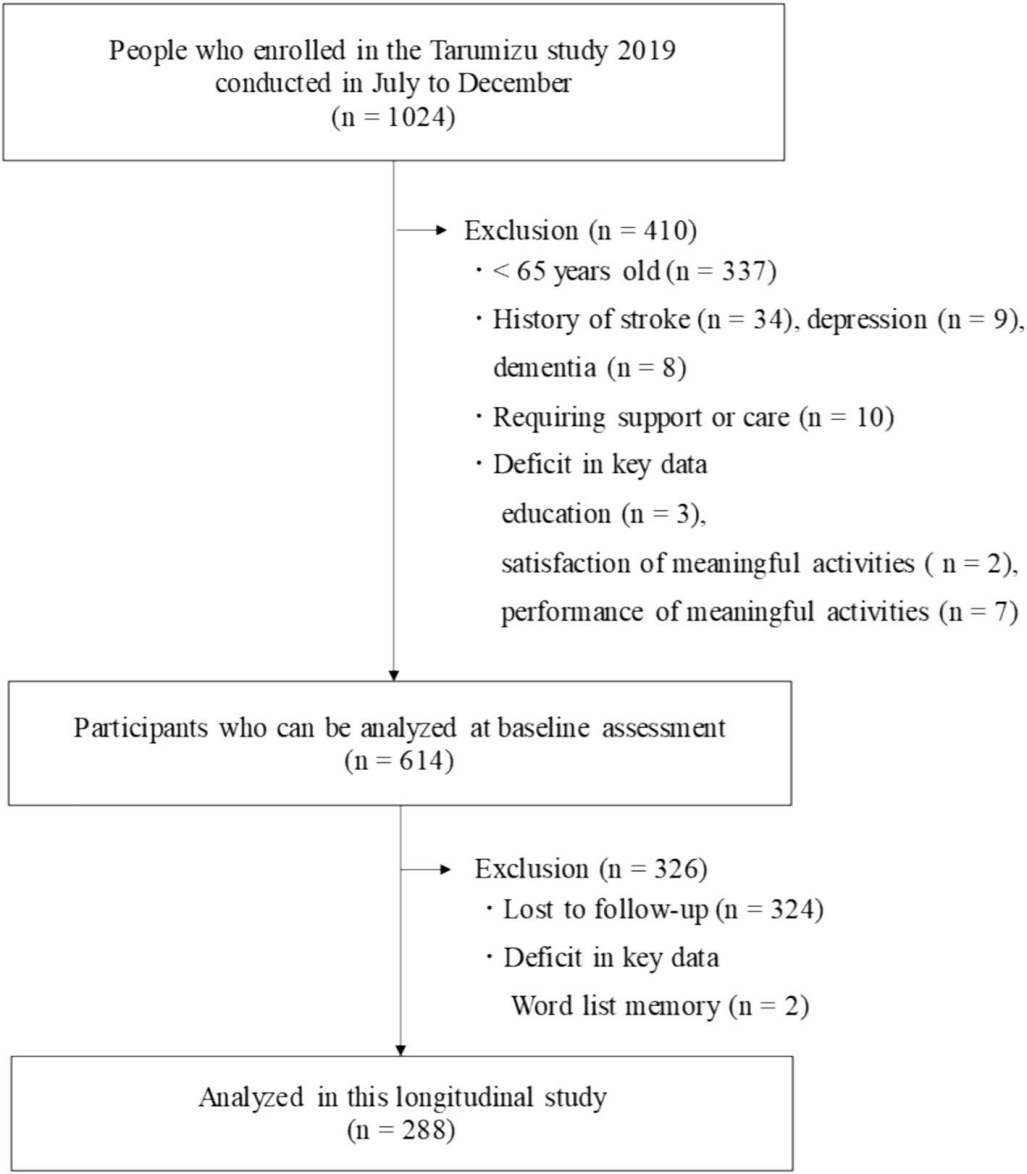
Flowchart of the inclusion and exclusion of the study participants

Community-dwelling older adults aged 65 years or older who participated in the Tarumizu Study (2019 and 2022) and underwent a longitudinal assessment of cognitive function were included in this study. The Tarumizu Study is a health assessment survey of Tarumizu residents aged 40 years and older conducted in collaboration with Tarumizu City Hall, Tarumizu Central Hospital, and Kagoshima University School of Medicine [19]. In the present study, the 2019 Tarumizu Study was used as the baseline survey, and a follow-up survey was conducted 3 years later in 2022. A total of 1024 people participated in the baseline study. People under the age of 65 (*n* = 337); with a history of stroke (*n* = 34), depression (*n* = 9), and dementia (*n* = 8); requiring support or care (*n* = 10), and missing primary data (educational history, satisfaction, and performance of meaningful activities) (*n* = 12) were excluded. This left 614 individuals. Of these 614 participants, those who did not attend the follow-up survey (*n* = 324) and those with missing primary data (word list memory) (*n* = 2) were excluded. Finally, data from 288 community-dwelling older adults aged 65 years and older (mean age 73.2 ± 5.5, 60.4% female) were analyzed longitudinally (Fig. 1).

### Outcome

#### Memory function

Memory function was assessed using the National Center for Geriatrics and Gerontology Functional Assessment Tool (NCGG-FAT) [20]. The NCGG-FAT is a tablet-based cognitive function testing tool developed by the National Institute for Longevity Sciences. Memory function was assessed by immediate recognition (word list memory—I) and delayed recall (word list memory—II) [20]. Specifically, participants were asked to memorize ten words displayed on a tablet device. Each of the ten target words was displayed for 2 s. Next, 30 words were displayed, including 10 target words and 20 distractor words, and participants were asked to immediately select the 10 target words. This task was repeated thrice. The average number of correct responses ranged between 0 and 10 (immediate recognition; word list memory—I). Additionally, participants were asked to recall the ten target words after approximately 20 min and write them down on paper. The total number of target words recalled was calculated: one point was awarded for each word correctly recalled within 60 s, with a maximum score of 10 (delayed recall; word list memory—II). Finally, the sum of the immediate recognition and delayed replay scores was used as the memory function score (score range 0–20) [20]. The first quintile of baseline memory function score (≤ 10.33 scores) was defined as poor memory function. In the supplement analysis, the first quintile of memory function score (≤ 9.33 score) was defined as poor memory function. The NCGG-FAT has good test reproducibility, moderate-to-high criterion-related validity, and predictive validity as a tool for assessing cognitive function in community-dwelling older adults [20, 21]. Memory function was assessed at the baseline and 3-year follow-up surveys as an outcome.

### Baseline assessment

#### Satisfaction with, and performance and frequency of meaningful activities

Meaningful activities were operationally defined as “activities that individuals consider important in their current daily lives” [10]. Meaningful activity choice, satisfaction, performance, and frequency were assessed using the Aid for Decision Making in Occupation Choice (ADOC) [22]. The ADOC comprises 95 activities and 8 categories (self-care, mobility, home life, work/education, interpersonal interaction, social life, sports, and leisure) related to “activity and participation” in the International Classification of Functional Living. Meaningful activities were assessed through face-to-face interviews using the ADOC, where participants were asked to select up to five meaningful activities and rank their importance. Meaningful activities were operationally defined as those ranked the most important among the five selected activities [10]. Satisfaction with meaningful activities was rated on a 5-point scale (1 = very dissatisfied, 5 = very satisfied), whereas performance was rated on a 10-point scale (1 = very difficult, 10 = perfect). In both analyses, overall satisfaction and performance were rounded down to the lowest 25% or less, with a satisfaction level of 4 or higher being the high-satisfaction group and 3 or lower being the low-satisfaction group. Performance of 8 or higher was considered the high-performance group and 7 or lower being the low-performance group [14]. The frequency of meaningful activities was determined by asking how often the participants engaged in meaningful activities (how many times a year, month, or week) and converting each frequency into times per week (times/week). Trained researchers, occupational therapy students, and occupational therapists performed the assessments.

#### Demographic and other variables

Demographic variables (age, sex, years of education, and living alone), number of daily medications, smoking, frequency of alcohol consumption (days/week) and medical history (hypertension, diabetes, and hyperlipidemia) were assessed through face-to-face interviews by a licensed physician or nurse. The years of education were classified into two groups: less and more than 12 years [23].

Weight (kg) and height (m) were measured, and body mass index (BMI) was calculated as weight divided by the square of height (kg/m^2^).

The questionnaire on hearing condition consisted of two questions: (1) Do you feel a disturbance in the conversation with the other person (conversation in a one person-to-one person situation)? and (2) Do you feel a disturbance in a conversation with several persons (conversation of one person with a group, such as conversation with four or five persons)? Participants were asked to respond to each of these two questions on a four-point scale (perfectly audible, almost audible, slightly audible, or disturbed). Hearing impairment was defined as “slightly audible” and/or “disturbed” in two conversation situations [24].

Physical function was assessed using maximum grip strength and normal walking speed. Grip strength was measured using a digital grip-strength meter (GRIP-D; Takei Scientific Instruments Co., Ltd., Niigata, Japan) to determine the maximum grip strength of the dominant hand. Normal walking speed was measured using a photoelectric sensor gait measuring device (YW; YAGAMI INC, Aichi, Japan). Acceleration and deceleration paths were set at 2 m before and after the flat 10 m measurement section, respectively, and the normal walking speed (m/s) was calculated. Referring to the Asian Working Group for Sarcopenia 2019, those with a maximum grip strength of less than 28 kg in men and 18 kg in women were defined as having muscle weakness. Those with a normal walking speed of less than 1.0 m/s were defined as having reduced walking speed. Finally, those with either muscle weakness or reduced walking speed were defined as having reduced physical function [25].

Depressive symptoms were assessed using the 15-item Geriatric Depression Scale (GDS-15) [26]. The GDS-15 is a 15-point scale with “yes” or “no” answers to 15 questions; the higher the total score on the GDS-15, the more depressed a person is. A cutoff score of ≥ 5, indicating the presence of depressive symptoms, was used [27].

### Statistical analysis

Participant characteristics are presented as mean ± standard deviation (SD) for continuous variables, population (%) for categorical variables, and median (interquartile range) for ordinal variables. Group comparisons between low and high satisfaction, and low and high completion groups for meaningful activities were analyzed using the unpaired t-test, Pearson’s χ-square test, Mantel–Haenszel tests for trend, and Mann–Whitney’s* U* test. Two-way repeated-measures analysis of covariance (ANCOVA) adjusted for age, sex, and educational history was used to analyze group (low- versus high-satisfaction group, low- versus high-execution group) and time interactions (baseline and three years later for memory function). All analyses were performed using IBM SPSS Statistics (version 29.0; IBM Japan, Tokyo, Japan), with a significance level of less than 5%.

## Results

Table 1 shows the group comparisons of baseline satisfaction with and performance of meaningful activities. In the between-group comparison of satisfaction with meaningful activities, depressive symptoms (*p* = 0.025) and the GDS-15 Score (*p* < 0.001) was statistically significantly lower in the high satisfaction group. In the between-group comparison of the performance of meaningful activities, the high-performance group had a higher BMI (*p* = 0.041), had a shorter follow-up survey time (*p* = 0.042), more frequent participation in meaningful activities (*p* = 0.002), a smaller proportion of individuals with depressive symptoms (*p* = 0.025), and a statistically significantly lower GDS-15 Score (*p* = 0.028). The between-group comparisons of satisfaction with and performance of meaningful activities revealed no significant differences in memory function (*p* > 0.05).

**Table 1:** Baseline participant characteristics

	All (*n* = 288)	Satisfaction			Performance		*p*-value
		Low satisfaction (*n* = 53)	High satisfaction (*n* = 235)	*p*-value	Low performance (*n* = 67)	High performance (*n* = 221)	
Age, years, mean ± SD	73.2 ± 5.5	72.5 ± 5.7	73.3 ± 5.5	0.290*	73.5 ± 5.7	73.1 ± 5.5	0.315*
Sex, female, *n* (%)	174 (60.4)	31 (58.5)	143 (60.9)	0.751^†^	44 (65.7)	130 (58.8)	0.559^†^
Education, 12 years or more, *n* (%)	199 (69.1)	37 (69.8)	162 (68.9)	0.901^†^	49 (73.1)	150 (67.9)	0.414^†^
Medications, *n*/day, mean ± SD	3.0 ± 3.0	2.9 ± 3.2	3.0 ± 3.0	0.799*	3.0 ± 3.0	3.0 ± 3.0	0.986*
Living alone, *n* (%)	76 (26.4)	17 (32.1)	59 (25.1)	0.298^†^	21 (31.3)	55 (24.9)	0.294^†^
BMI, kg/m^2^, mean ± SD	23.0 ± 3.1	22.3 ± 3.3	23.2 ± 3.1	0.052*	22.3 ± 3.4	23.2 ± 3.0	0.041*
Smoking history, yes, *n* (%)	85 (29.5)	17 (32.1)	68 (28.9)	0.651^†^	18 (26.9)	67 (30.3)	0.587^†^
Frequency of alcohol consumption, days/week, *n* (%)				0.555^‡^			0.364^‡^
No drinking	177 (61.5)	30 (56.6)	147 (62.6)		44 (65.7)	133 (60.2)	
1–6 days	39 (13.5)	9 (17.0)	30 (12.8)		9 (13.4)	30 (13.6)	
Everyday	72 (25.0)	14 (26.4)	58 (24.7)		14 (20.9)	58 (26.2)	
Hearing impairment, *n* (%)	38 (13.2)	8 (15.4)	30 (12.8)	0.614^†^	10 (15.2)	28 (12.7)	0.602^†^
Hypertension, *n* (%)	129 (44.8)	24 (45.3)	105 (44.7)	0.937^†^	32 (47.8)	97 (43.9)	0.577^†^
Diabetes, *n* (%)	37 (12.8)	7 (13.2)	30 (12.8)	0.931^†^	8 (11.9)	29 (13.1)	0.800^†^
Hyperlipidemia, *n* (%)	83 (28.8)	13 (24.5)	70 (29.8)	0.445^†^	22 (32.8)	61 (27.6)	0.407^†^
Follow-up period, days, mean ± SD	1167.5 ± 78.1	1164.8 ± 74.2	1168.1 ± 79.1	0.783^†^	1184.5 ± 76.3	1162.3 ± 78.1	0.042^†^
Frequency of meaningful activities, day/week, median (IQR)	7.0 (1.0—7.0)	5.0 (1.0—7.0)	7.0 (1.4—7.0)	0.308^§^	3.0 (1.0—7.0)	7.0 (2.0—7.0)	0.002^§^
Poor physical function, *n* (%)	58 (20.3)	12 (22.6)	46 (19.7)	0.636^†^	12 (17.9)	46 (21.0)	0.582^†^
Depressive symptoms, *n* (%)	51 (17.7)	15 (28.3)	36 (15.3)	0.025^†^	18 (26.9)	33 (14.9)	0.025^†^
GDS-15, score, mean ± SD	2.6 ± 2.4	3.7 ± 2.6	2.4 ± 2.2	< 0.001*	3.2 ± 2.3	2.5 ± 2.4	0.028*
Poor memory function, *n* (%)	53 (18.4)	8 (15.1)	45 (19.1)	0.491	13 (19.4)	40 (18.1)	0.809
Memory function, score, mean ± SD	12.9 ± 2.9	12.7 ± 2.7	12.9 ± 3.0	0.312*	12.8 ± 3.2	12.9 ± 2.8	0.762*

Missing data, Poor physical function (*n* = 2), Body mass index (*n* = 5), Hearing loss (*n* = 1)

*SD* standard deviation, *IQR* interquartile range, *BMI* Body mass index, *GDS-15* Geriatric Depression Scale-Short Version

*Student’s *t*-test

^†^Pearson’s *χ*^2^ test

^‡^Mantel–Haenszel tests for trend

^§^Mann–Whitney *U* test

Supplementary Table 1 shows the group comparisons between the participants who could not be followed up and those included in the final analysis. Participants included in the final analysis were younger, were more educated, had fewer poor physical function, had fewer poor memory function, and had statistically significantly higher memory function scores (*p* < 0.001) than those who were not followed-up.

**Fig. 2:**
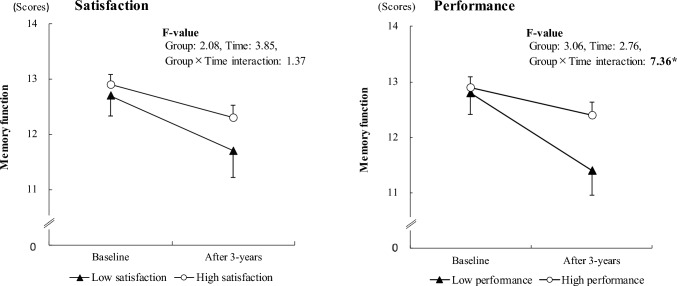
Satisfaction with and performance of meaningful activities, and age-related changes in memory function (ANCOVA for repeated measures, covariance; adjusted with age, sex, and education). * *p* < 0.01

Figure 2 shows the change in memory function between the baseline and follow-up surveys, and results of the repeated-measures ANCOVA (adjusted variables: age, sex, and years of education). Between groups in satisfaction with meaningful activities and changes in memory function scores were 12.7 ± 2.7 at baseline and 11.7 ± 3.5 at 3 years for the low satisfaction group, and 12.9 ± 3.0 at baseline and 12.3 ± 3.5 at 3 years for the high satisfaction group. The between-group and time main effects, and between-group interactions with memory function were not significant across the groups for satisfaction with meaningful activities (*F* = 1.37, *p*  = 0.24). Changes between groups in performance of meaningful activities and memory function scores were 12.8 ± 3.2 at baseline and 11.4 ± 3.6 at 3 years for the low performance group, and 12.9 ± 2.8 at baseline and 12.4 ± 3.5 at 3 years for the high satisfaction group. There were no significant group or time main effects with memory function across groups for the performance of meaningful activities, but the group interaction was significant (*F* = 7.36, *p* = 0.007).

**Fig. 3:**
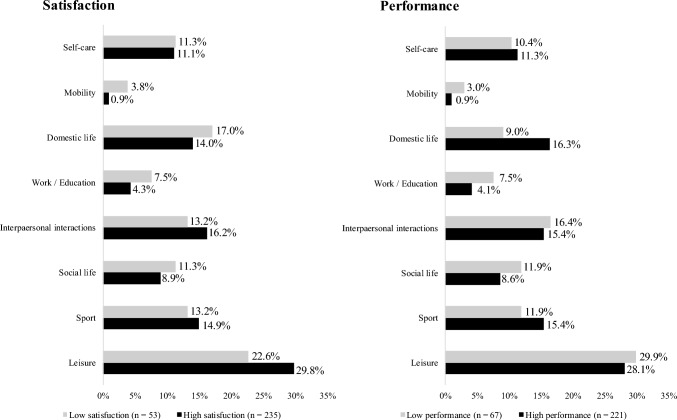
The percentages of satisfaction and performance activity categories for meaningful activities in baseline. No significant difference between the two groups in satisfaction and performance (satisfaction group: *p* = 0.63, performances group: *p* = 0.56)

Figure 3 shows the characteristics of baseline satisfaction with and performance of meaningful activities between the groups and categories. There were no significant associations between the groups and categories of satisfaction with and performance of meaningful activities (satisfaction group, *p* = 0.63; performance group, *p* = 0.56).

## Discussion

This study examined the effects of satisfaction with and performance of meaningful activities on memory function three years later among community-dwelling older adults. The results suggest that higher performance of meaningful activities may be more protective against changes in memory function three years later than lower performance. However, there was no significant relationship between satisfaction with meaningful activities and memory function.

Interpretations in terms of socioemotional selectivity and self-determination theories are useful for explaining the association between meaningful activities and memory function [28, 29]. According to socioemotional selectivity theory, as people age, they invest more resources in experiences that are “personally” emotionally fulfilling (enjoyable, goal-related, etc.) [30]. Furthermore, since meaningful activities are self-selected by individuals, they may be more likely to be intrinsically motivated rather than when involved in externally provided activities. According to the motivation for human behavior (self-determination theory), intrinsic motivation is considered more autonomous in goal-directed behavior than extrinsic motivation [31]. Furthermore, participation in meaningful activities may be related to basic human psychological needs (autonomy, competence, and relatedness) [32]. That is, meaningful activities are self-selected, emotionally fulfilling experiences [28], and are presumed to be activities that are likely to continue autonomously.

Supporting these claims, the median frequency of participation in meaningful activities (times/week) in this survey was 7 (times/week) overall, indicating older adults' daily participation in meaningful activities. Furthermore, the median frequency of meaningful activities was 3 and 7 times/week for the low- and high-performance groups, respectively. Clearly, the group with high performance in meaningful activities participated in meaningful activities more frequently than did the group with low performance.

Research suggests that engagement in meaningful activities is associated with the anterior cingulate cortex, which is associated with memory and motivation [16]. This suggests that high performance of meaningful activities may be associated with greater stimulation of cognitive activation from meaningful activities than low performance. This can be one possible explanation for the possibility that high performance of meaningful activities may be more protective against cognitive decline than low performance of meaningful activities.

Satisfaction with meaningful activities has no significant effect on cognitive function changes. This may be because satisfaction with meaningful activities is a particularly subjective indicator [12, 22]. Since satisfaction reflects only subjective feelings, whether it reflects the actual frequency or activity status is unclear. Next, satisfaction with and frequency of meaningful activities did not significantly differ. Thus, frequency may not be directly related with satisfaction with meaningful activities. Furthermore, research reveals no association between meaningful activity (occupation) efforts at a single point and the activity of the dopaminergic neural pathways (involved in motivation) in the brain, as assessed using functional magnetic resonance imaging [33, 34].

These findings suggest that, among community-dwelling older adults, satisfaction with meaningful activities may have characteristics which make it less responsive to stimulating cognitive function derived from ongoing engagement in meaningful activities (i.e., the opportunity to gain emotionally fulfilling experiences). This may be one explanation for the lack of a significant effect of satisfaction with meaningful activities on changes in cognitive function.

The relationship between meaningful activities and depressive symptoms should also be considered when interpreting the results of this study [35]. The National Institute for Health and Care Excellence has shown that “participation in meaningful activities” is a quality indicator of mental health in older adults living in nursing homes [36]. In support of these claims, a higher proportion of those in the low satisfaction and performance of meaningful activities group had depressive symptoms compared to those in the high satisfaction and performance group. This suggests that low activity owing to depressive symptoms may explain lower satisfaction with and performance in meaningful activities [37]. Therefore, depressive symptoms may need to be considered when interpreting the relationship between satisfaction, performance, and memory function in meaningful activities.

A strength of this study is that the categories of meaningful activities were not related with satisfaction or performance. This result appropriately reflects the concept of meaningful activities. A previous study reported that self-selection of activities of interest activate midline frontal electroencephalography compared with self-selection or forced-choice conditions of disliked activities [38]. Furthermore, engagement in personally meaningful activities has been suggested to be associated with memory function in cognitively healthy older adults [5]. These findings suggest that examining involvement that enhances the performance of activities that are meaningful “to the individual” rather than just focusing on specific activities may be an important perspective in preventing memory function decline.

This study has several limitations. First, given the baseline group comparison results between participants who were not followed up and those who were, the included participants may reflect the characteristics of community-dwelling older adults with better health status. Consequently, age-related changes in cognitive function may have been underestimated because of survival effects. Second, we did not sufficiently adjust for potential confounders (e.g., physical activity and social participation) between satisfaction with and performance of meaningful activities, and changes in memory. Third, between the baseline (2019) and follow-up surveys (2022), the government’s restrictions on activities due to the COVID-19 pandemic and impact of contracting the coronavirus on cognitive function may have influenced the results. However, we did not consider these aspects [39]. Fourth, although satisfaction with meaningful activities did not affect memory function among community-dwelling older adults, this result should be interpreted cautiously, as memory function was determined only by immediate recapture and delayed replay. To better interpret the relationship between satisfaction with meaningful activities and cognitive function, scholars should examine the relationship between meaningful activities, and overall cognitive function and other cognitive domains, considering participant characteristics (disease, age, sex, etc.).

## Conclusion and implications

Regardless of the meaningful activity category, the results suggest that higher performance of meaningful activities may be a protective factor against age-related changes in memory function. However, satisfaction with meaningful activities was not significantly related. Health support measures against memory decline should carefully assess meaningful activities that reflect individual needs and characteristics, and foster involvement that enhances performance.

## Supplementary Information

Below is the link to the electronic supplementary material.

Supplementary file1 (DOCX 19 KB)

## Data Availability

The data supporting the findings of this survey cannot be shared for privacy reasons. Further enquiries can be directed to the corresponding author.
