# Supplementary material for: Averting older adults' memory function decline via meaningful activities: a follow-up longitudinal study
Source: Eur Geriatr Med. 2024 Sep 6;15(6):1793–801. doi: 10.1007/s41999-024-01044-4 (PMC11632033; doi:10.1007/s41999-024-01044-4)
Supplement: Supplementary file 1 — Supplementary file1 (DOCX 19 KB) [file 41999_2024_1044_MOESM1_ESM.docx]

| Supplementary Table 1. Baseline characteristics of participants included in the analysis and those lost to follow-up survey. | | | | |
| --- | --- | --- | --- | --- |
|  | All (n = 612) | Participants who could not follow-up (n = 324) | Study population (n = 288) | p-value |
| Age, years, mean ± SD | 74.1 ± 6.3 | 74.9 ± 6.9 | 73.2 ± 5.5 | < 0.001^*^ |
| Sex, female, n (%) | 388 (63.4) | 214 (66.0) | 174 (60.4) | 0.149^†^ |
| Education, 12 years or more, n (%) | 375 (61.3) | 176 (54.3) | 199 (69.1) | < 0.001^†^ |
| Medications, n/day, mean ± SD | 3.2 ± 3.0 | 3.3 ± 3.0 | 3.0 ± 3.0 | 0.278^†^ |
| Living alone, n (%) | 174 (28.4) | 98 (30.2) | 76 (26.4) | 0.291^†^ |
| BMI, kg/m^2^, mean ± SD | 23.1 ± 3.5 | 23.1 ± 3.4 | 23.0 ± 3.1 | 0.670^*^ |
| Smoking history, yes, n (%) | 176 (28.8) | 91 (28.1) | 85 (29.5) | 0.697^†^ |
| Frequency of alcohol consumption, days/week, n (%) |  |  |  | 0.052^‡^ |
| No drinking | 396 (64.7) | 219 (67.6) | 177 (61.5) |  |
| 1 to 6 days | 85 (13.9) | 46 (14.2) | 39 (13.5) |  |
| Everyday | 131 (21.4) | 59 (18.2) | 72 (25.0) |  |
| Hearing impairment, n (%) | 82 (13.4) | 44 (13.6) | 38 (13.2) | 0.890^†^ |
| Hypertension, n (%) | 293 (47.9) | 164 (50.6) | 129 (44.8) | 0.150^†^ |
| Diabetes, n (%) | 79 (12.9) | 42 (13.0) | 37 (12.8) | 0.966^†^ |
| Hyperlipidemia, n (%) | 176 (28.8) | 93 (28.7) | 83 (28.8) | 0.975^†^ |
| Poor physical function, n (%) | 171 (28.3) | 113 (35.4) | 58 (20.3) | < 0.001^†^ |
| Depressive symptoms, n (%) | 122 (20.0) | 71 (22.0) | 51 (17.7) | 0.181^†^ |
| GDS-15, score, mean ± SD | 2.8 ± 2.5 | 2.9 ± 2.6 | 2.6 ± 2.4 | 0.143^*^ |
| Poor memory function, n (%) | 118 (19.3) | 81 (25.0) | 37 (12.8) | < 0.001^†^ |
| Memory function, score, mean ± SD | 12.4 ± 3.3 | 11.9 ± 3.6 | 12.9 ± 2.9 | < 0.001^*^ |
| Meaningful activities status | | | |  |
| High satisfaction, n (%) | 504 (82.4) | 269 (83.0) | 235 (81.6) | 0.644^†^ |
| High performance, n (%) | 453 (74.0) | 232 (71.6) | 221 (76.7) | 0.149^†^ |
| SD, standard deviation; BMI, Body mass index; GDS-15, Geriatric Depression Scale-Short Version. | | | | |
| Missing data, Medications (n = 1), BMI (n = 9), Hearing loss (n = 2), Poor physical function (n = 7), GDS-15 (n = 2). | | | | |
| * Student’s t-test, † Pearson’s χ^2^ test, ‡ Mantel-Haenszel tests for trend. | | | | |
